# Examining the influence of substance use on mental health rating during COVID-19: A Canadian perspective

**DOI:** 10.3389/fepid.2023.1067492

**Published:** 2023-03-01

**Authors:** Yadurshana Sivashankar, Ze Lin Chen

**Affiliations:** Department of Psychology, Faculty of Arts, University of Waterloo, Waterloo, ON, Canada

**Keywords:** COVID-19, mental health, substance use, social determinants, sex differences, ordered logistic regression

## Abstract

**Introduction:**

Substance use and mental health symptoms (e.g., depression and anxiety) have increased during the COVID-19 pandemic, partly due to implementation of physical distancing measures aimed at containing the spread of the SARS-CoV-2 virus. However, there is limited pandemic-specific research that has examined the relationship between substance use and mental health with other correlates of well-being, including life satisfaction and social confidants.

**Methods:**

In the current study, we used ordered logistic regression analyses to examine whether a greater frequency of substance use (e.g., alcohol, cannabis, and opioids) during the pandemic predicted poorer ratings of self-reported mental health in a large sample of Canadians aged 15 to 64 years. We further considered whether life satisfaction and number of social confidants interacted with substance use to influence mental health, and stratified the models by sex and personal feelings of shame surrounding the use of substances (i.e., high and low shame).

**Results:**

Findings indicated that frequency of substance use was significantly associated with increased odds of reporting poorer mental health for males and females exhibiting both low and high shame. In females reporting low shame, we found that as frequency of cannabis use increased, life satisfaction has a much greater positive association with mental health. Whereas, in females disclosing high shame, maintaining social relations was particularly important to benefit the mental health of current users of opioids, relative to past and non-users. No such interaction was found in males.

**Discussion:**

Overall, the findings of the current study showed the negative mental health consequences of substance use during COVID-19 in a large Canadian sample, and most importantly revealed a critical sex difference in the way in which social determinants interact with substance use to influence mental health.

## 1. Introduction

The spread of the coronavirus disease (COVID-19) has emerged as a global pandemic, and has had a profound impact on the existing economic, social, and political landscape of communities around the world (1). For example, COVID-19 outbreak has led to fewer social interactions due to implementation of social distancing measures, a reduction in employment opportunities, and limited accessibility to social and health services (1). Another adverse outcome of the COVID-19 pandemic is the psychological distress experienced by public in response to social restrictions (2, 3). For instance, a survey conducted by Wang and colleagues (4) examined the psychological outcomes of early stages of COVID-19 restrictions among the Chinese population. They showed that nearly half of the sample (1,211 participants) in their data rated the mental health distress of the outbreak to be moderate or severe. Another survey conducted on university students in China showed that anxiety was a common concern experienced among students due to the pandemic, and family-income, living with parents, and overall social support served as protective factors against anxiety (5). Since these early studies, a similar trend (an increase in self-reported levels of depression and anxiety during COVID-19) has been reported in surveys conducted in Italy (6), Spain (7), Germany (8), India (9), United States (10), and Canada (11). Solomou and Constrantinidou (12) particularly emphasized the crucial role of individual and social contextual differences when evaluating the severity of mental health symptoms during COVID-19. For example, they observed that women, younger adults (18–29), students, and those with prior psychiatric history, were at a higher risk to experience depressive and anxiety symptoms during the pandemic. It is also possible that implementation of social distancing measures have pushed people to consume drugs, such as alcohol, cannabis, and opioids, that evoke a sedative effect to cope with more time spent indoors in solitude (13). Finally, both rates of transmission and mortality from the SARS-CoV-2 virus has been higher among males than in females (14). Such critical differences in virus contraction and remission between both sexes could reflect differences in maladaptive coping strategies, such as, the consumption of substances, and subsequent poorer outcomes in mental health (15). Thus, in the current study, we stratified our analyses by sex to determine any group differences pertaining to the influence of substance use on mental health ratings.

In light of these results, the objective of the current study was to examine whether the frequency of substance use (e.g., alcohol, cannabis, and opiates) during COVID-19 significantly predicted ones’ perceived mental health. We further considered whether life satisfaction and number of social confidants (i.e., the number of social connections one has maintained over the course of the pandemic) interacted with substance use to influence mental health ratings. In this cross-sectional study, we used the “Substance Use and Stigma During the Pandemic” dataset from the Canadian Perspective Survey Series 2021 from Statistics Canada (16). The use of this dataset allowed us to examine the influence of substance use on mental health in a large Canadian sample, with the aim of offering novel insight about the critical relationship between substance use and mental health within the context of COVID-19.

### 1.1. Link between substance use and perceived mental health

Past studies have established a significant association between substance use and mental health symptoms such as depression and anxiety [see (17) for review]. In the context of COVID-19, we hypothesized substance use to be a critical predictor of mental health as long periods of home confinement could have prompted individuals to consume substances as a means to cope with social isolation and loneliness (13, 18). In addition, limited accessibility to legal, health and social services during national lockdowns in Canada may have further exacerbated the use of substances as coping mechanisms (19). It is also worthwhile to note that governing agents in many countries around the world, including Canada, deemed substances such as alcohol and tobacco as “essential commodities” to be sold during lockdowns (18). Thus, we believe that the unique social situation presented by COVID-19 could have encouraged a greater frequency of substance use among Canadians relative to pre-pandemic.

### 1.2. The role of life satisfaction and social confidants

In addition to assessing the influence of substance use on perceived mental health (outcome variable), we also sought to examine the interaction between life satisfaction (measured on a 11 point Likert-scale ranging from 0 to 10) and social confidants (measured as ordered levels with options “None”, “One or two”, “Three to five”, “Six to nine”, “Ten or more.”) with substance use on our outcome. Prior research suggests that self-reported life satisfaction is measured in relative to one's global cognitive functioning and achievements obtained across a broad range of human activities at school, work, family, and social relations (20). Previous studies have reported life satisfaction to be a significant predictor of how well an individual optimally responds to life's stressors [(21, 22); see (23) for review]. Another social factor considered in our analysis was the number of family and friends an individual feels connected to during the pandemic, denoted as social confidants in the current study. Past studies support the view that imposed social isolation inflicts considerable psychological harm to people (24). Thus, it is beneficial for one to maintain social connections, even if it is virtual, to overcome the mental burden of social isolation (25). Thus, individuals who use substances in greater frequency, and as a result report poorer mental health ratings, might particularly benefit from social relations and having greater life satisfaction to alleviate mental distress.

### 1.3. Current study

In our current study, we hypothesized substance use (measured by frequency of alcohol, cannabis, and opioid use) to be negatively associated with one's subjective reporting of their mental health (outcome variable). That is, we were particularly interested in predicting whether an increase in the frequency of substance use, irrespective of the type of substance, resulted in an increase in the odds of reporting poorer mental health. On the other hand, we hypothesized greater life satisfaction and number of social confidants to increase the odds of reporting better mental health. Specifically, the influence of life satisfaction and social confidants on mental health was predicted to differ based on the frequency of substance use during the pandemic. Further, we stratified our analyses by sex and personal feelings of shame and guilt surrounding general substance use, since we predicted these variables to distinctively influence the association between substance use and perceived mental health. Shame is conceptualized as an intense negative emotion resulting in feelings of inferiority and powerlessness (26). For example, past research suggests that in adults, shame has been strongly implicated in behaviors that allow individuals to escape feelings of loneliness and failure, such as, sexual risk-taking, binge eating, and substance use (26, 27). Another important distinction is that females seeking treatment for substance use face greater stigma than males, often risking the loss of intimate relationships, as well as the custody of children (28). Therefore, females who participate in treatment programs for substance use often experience enhanced shame and guilt compared to males (28). Further, there are critical sex differences between men and women at all stages of substance consumption, that is, at initial use, maintenance, withdrawal, and relapse [see (15) for review]. For example, women experience a stronger pleasurable response to drugs than men do, and are more likely to self-medicate than men. Whereas, men are more likely to use substances to engage in risky behavior or to be associated with a particular social group. Similarly, women tend to progress more rapidly than men from initial use, and are more prone to experience stronger withdrawal symptoms [See (15) for review]. The differences in the manner in which men and women initiate and sustain substance use could also distinctively influence mental health outcomes in both sexes. In light of these findings, we stratified our analyses by both sex and personal feelings of shame and guilt to detect any possible group differences on our outcome measure. Finally, education and employment status served as covariates in our model, as past research suggests these variables to be significant contextual social differences linked to mental health during the COVID-19 pandemic (12).

## 2. Materials and methods

### 2.1. Dataset

The current study used the “Canadian Perspectives Survey (CPSS) Series 6, 2021: Substance Use and Stigma During the Pandemic” dataset from Statistics Canada to examine whether substance use predicted mental health reporting (16). The data was collected from a cross-sectional survey administered to target residents (15 years of age or older) of the 10 Canadian provinces. The survey included questions on socio-economic background, mental health, social interactions, utilization of services, and the frequency of use of alcohol, cannabis, opioids, and non-prescription substances during the pandemic. The survey was distributed to a randomly selected population using stratified, multi-stage probability sampling design^1^. The original dataset included a sample of 3,941 participants. Surveys with a missing response (i.e., “not stated”) to covariate, independent, or dependent variables were omitted from the analysis, resulting in a final sample size of 3,790 (see notes under Table 1 for exclusion criteria).

**Table 1:** Sample characteristic*s*.

	Low Shame (*n* = 1698)	High Shame (*n* = 2092)
**Stratification and Ordinal variables, *n* (%)**		
Stratification variable		
Sex		
Male	608 (36%)	1,120 (54%)
Female	1,090 (64%)	972 (46%)
Covariates		
Employment		
Not employed	689 (41%)	830 (40%)
Employed but absent for reasons related to COVID	21 (1%)	38 (2%)
Employed but absent for reasons not related to COVID	43 (3%)	41 (2%)
Employed and at work	945 (56%)	1,183 (57%)
Education		
Less than high school	77 (5%)	104 (5%)
High school diploma or trade certificate	304 (18%)	393 (19%)
College or other non-university degree	545 (32%)	692 (33%)
University degree that is below Bachelor's or Bachelor's degree	504 (30%)	611 (29%)
Above Bachelor's degree	268 (16%)	292 (14%)
Predictors		
Alcohol use		
Non-user	655 (39%)	524 (25%)
Past user	783 (46%)	784 (37%)
Light user	169 (10%)	410 (20%)
Moderate user	47 (3%)	137 (7%)
Heavy user	44 (3%)	237 (11%)
Cannabis use		
Non-user	1,318 (78%)	1,384 (66%)
Past user	243 (14%)	253 (12%)
Light user	70 (4%)	151 (7%)
Moderate user	28 (2%)	129 (6%)
Heavy user	39 (2%)	175 (8%)
Opioid use		
Non-user	1,367 (81%)	1,603 (77%)
Past user	257 (15%)	346 (17%)
Current user	74 (4%)	143 (7%)
Number of social confidants		
None	55 (3%)	110 (5%)
1 or 2	543 (32%)	829 (40%)
3 to 5	692 (41%)	726 (36%)
6 to 9	238 (14%)	237 (11%)
More than 10	170 (10%)	154 (7%)
**Continuous variables, *M* (*SD*)**		
Predictor		
Life satisfaction	7.80 (2.11)	7.47 (2.23)
Outcome		
Perceived mental health	3.41 (1.01)	3.21 (1.07)

*Note.* Surveys with missing responses to any of the items of interest were excluded. The number of excluded responses for each item is as follows: alcohol use (*n* = 3), cannabis use (*n* = 2), opioid use (*n* = 4), social confidants (*n* = 1), life satisfaction (*n* = 3), perceived mental health (*n* = 7).

The independent variables of the current study included education and employment status serving as covariates, and life satisfaction, number of social confidants, alcohol use, cannabis use, and opioid use, serving as predictors. Self-rating of perceived mental health was the outcome variable. Education, employment status, social confidants and substance use variables were recoded to reflect ordered levels. That is, for these variables, participants were grouped in an ordinal manner based on their responses. For example, participants who had never used opioids were coded as 0, participants who did not use opioids in the past 30 days were coded as 1 and were classified as past users, and respondents who answered “Yes” were current users, and they were coded as 2. Hence, these variables were ordinal in nature. Life satisfaction and self-rating of mental health were continuous variables.

### 2.2. Measures

#### 2.2.1. Covariates

Covariates in the study were education and employment status. Each of the variables were measured using one survey question. Employment status was measured as a set of categories including “not employed”, “employed and at work at least part of the reference week”, “employed but absent from work for reasons not related to COVID-19”, and “employed but absent from work due to COVID-19”. Unemployment was coded as 0. “Employed but absent from work due to COVID-19” was coded as 1. “Employed but absent from work not due to COVID-19” was coded as 2. “Employed and at work” was coded as 3.

Education status was operationalized by five levels. The first level was coded as 0, it consisted of the group who obtained “less than high school diploma or its equivalent”. The second level was coded as 1. It consisted of categories including “High school diploma or a high school equivalency certificate” and “Trade certificate or diploma”. The third level was coded as 2. It consisted of people who obtained “College/CEGEP/other non-university certificate or diploma” or “University certificate or diploma below the bachelor's level”. The fourth level was coded as 3, it consisted of people who obtained “Bachelor's degree (e.g., B.A., B.Sc., LL.B.).” The fifth level was coded as 4, it consisted of people who obtained “University certificate, diploma, degree above the BA level”.

#### 2.2.2. Predictors

Frequency of alcohol use was measured by one item that assessed how many times the individual had 5 or more drinks on one occasion in the past 30 days. The levels of this variable were “4 times a week or more”, “2 or 3 times a week”, “once a week”, “2 to 3 times in the past 30 days”, “Once in the past 30 days”, “Not in the past 30 days”. People who never had alcohol were coded as 0. People who did not have 5 or more drinks in the past 30 days were classified as past alcohol users and were coded as 1. Participants who had 5 or more drinks once or two to three times in the past 30 days, were aggregated into one group (we classified this group as “light alcohol users”) and were coded as 2. The group of moderate users included those who had 5 or more drinks once a week, and were coded as 3. Participants who had five or more drinks 2 or 3 times a week, or 4 times a week or more were classified to be heavy alcohol users and were coded as 4.

Frequency of cannabis use was measured by one item, which assessed how many days the individual had used cannabis in the past 30 days. The levels of this variable were “Never used cannabis”, “No, not during the past 30 days”, “1 day in the past 30 days”, “2 or 3 days in the past 30 days”, “1 or 2 days per week”, “3 or 4 days per week”, “5 or 6 days per week”, “Daily.” Participants who never used cannabis were given a code of 0. Participants who did not use cannabis in the past 30 days were coded as 1 and were classified as past cannabis users. Light cannabis users consisted of those who had cannabis either once, or two to three times, in the past 30 days. This group was coded as 2. Moderate cannabis users included those who used cannabis up to four days per week. They were coded as 3. Heavy cannabis users were those who used cannabis either five or six days per week or daily. They were coded as 4.

Opioids use in the past 30 days was measured by one item, which asked whether the individual had used drugs containing opioids, prescribed or not, in the past 30 days. The levels of this variable were “Yes”, “Not during the past 30 days”, and “Never used opioid products”. Participants who had never used opioids were coded as 0. Participants who did not use opioids in the past 30 days were coded as 1 and were classified as past users. Respondents who answered “Yes” were current users, and they were coded as 2.

Number of social confidants was measured using one question which asked, “Approximately how many relatives and friends do you have who you feel close to, that is, who you feel at ease with and can talk to about what is on your mind?” The options included “None”, “One or two”, “Three to five”, “Six to nine”, “Ten or more.” Each level was represented by a value from 0 to 4 that corresponded with increasing numbers of social confidants.

Life satisfaction was measured using one item “How do you feel about your life as a whole right now?” This variable was rated using a 11-point Likert scale, ranging from “0-very dissatisfied” to “10-very satisfied”.

#### 2.2.3. Stratification

We stratified our data by sex (i.e., groups of males and females). We also stratified the dataset by shame. Shame was measured on the basis of agreeing to the following statements about substance use in general: “Problem with alcohol/drugs, embarrassed to tell friends/family”, “Alcohol/drug problem, embarrassing to seek help/treatment”, “Embarrassing to tell friends/family about my alcohol/drug use”, “Embarrassing to seek help/treatment for my alcohol/drug use”, “Scared how people will react if they find out about my alcohol/drug use”, and “Need to hide my problems with alcohol/drugs from my friends/family”. These items were rated using a Likert scale. Cronbach's alpha between these items was 0.82; indicating good internal consistency among the items for the construct (shame) measured (29). An average composite score was calculated for each individual. Using a median split, people who scored above the median was classified as “high shame”, and anyone who scored below the median was classified as “low shame”.

#### 2.2.4. Outcome

The outcome of our analytical model was perceived mental health. This was measured using the question: “In general, how would you describe your mental health?” This variable was rated using a 5-point Likert scale, from 0 to 4, with higher values indicating better mental health.

### 2.3. Statistical analysis

We stratified our sample by sex and feelings of shame towards general substance use and conducted an ordered logistic regression analysis to assess whether frequency of substance use influenced mental health differentially across these groups. We added variables of interest into our model in a hierarchical manner. In the first step, employment, education, and the frequency of alcohol, cannabis, and opioid use were entered into the model. In the second step, we sought to assess the association between social confidants and life satisfaction with our outcome measure, while controlling for the covariates and substance use variables. In the third step, we entered interaction terms between each substance use variable and social determinants of well-being to evaluate whether life satisfaction and social confidants interacted with substance use to influence mental health.

All models were estimated using the “*polr*” function in the MASS package [version 7.3.57 (30);] using R 4.1.3 (31). Statistical tests were performed to test the assumptions of ordinal regression. Multi-collinearity between independent variables was examined by computing inter-variable correlations and variance inflation factors (VIF). All VIF values of the predictor variables fall below 2, indicating low multicollinearity. The assumption of proportional odds was tested using a *χ*2 test using the “*vglm”* function from the VGAM package [version 1.1.6 (32);]. Separate models were created with and without the proportional odds assumption. The deviances and degrees of freedom of the models were entered into a *χ*2 test. The result indicated that there was not a significant difference between the Akaike information criterion of the models. Thus, the proportional odds assumption was not violated.

## 3. Results

### 3.1. Sample descriptives

The sample was stratified by sex and feelings of shame towards substance use. Thus, resulting in groups of females with high shame, females with low shame, males with high shame and males with low shame (see Table 1 for demographic characteristics). Parallel analyses were run for each group.

### 3.2. Ordered logistic regression results

#### 3.2.1. People reporting lower levels of shame towards substance use

##### 3.2.1.1. Males

For males reporting low levels of shame towards general substance use, only cannabis use was significantly associated with mental health (Step 1; see Table 2). That is, increase in one level of frequency of cannabis use was associated with a 24% increase in the odds of reporting poorer mental health (OR = 0.76, *p* = .002). Opioid and alcohol use did not significantly correlate with mental health reporting (*ps* > .122; *ps* > .391 respectively) for males exhibiting low shame towards substance use. In Step 2 of our analyses, we entered life satisfaction and social confidants to examine their influence on our outcome measure, above and beyond education, employment status, and frequency of substance use. Life satisfaction significantly correlated with mental health, such that one unit increase in life satisfaction was associated with 2.14 times increase in the odds of reporting better mental health, *p* < .001. Number of social confidants was also significantly associated with better mental health reporting (OR = 1.61, *p* < .001). As in Step 1, an increase in the frequency of cannabis use (OR = 0.80, *p* = .016) was significantly associated with increased odds of poorer mental health reporting, and this pattern was not true for both alcohol and opioid intake. In Step 3 of our analyses, we probed for the interaction between life satisfaction and social confidants with frequency of substance use on one's perceived mental health. The interaction terms between life satisfaction and the various substances were not significant for this sub-group (see Step 3 in Table 2). However, the association between life satisfaction and social confidants with mental health reporting remained to be highly significant in Step 3 (OR = 2.25, *p* < .001 for life satisfaction; OR = 1.53, *p* = .001 for social confidants), as in Step 2.

**Table 2:** Regression model for people reporting low feelings of shame towards substance us*e.*

	Males			Females		
Predictors	OR	95% CI	*p*	OR	95% CI	*p*
**Step 1**	R^2^ Nagelkerke = 0.042			R^2^ Nagelkerke = 0.039		
Employment	0.87	0.79–0.97	**0** **.** **009**	0.88	0.81–0.95	**0** **.** **001**
Education	1.14	1.00–1.30	**0** **.** **043**	1.06	0.95–1.17	0.307
Alcohol use	1.07	0.92–1.25	0.391	1.07	0.94–1.22	0.291
Cannabis use	0.76	0.64–0.90	**0** **.** **002**	0.76	0.66–0.87	**<0** **.** **001**
Opioid use	0.80	0.59–1.06	0.122	0.70	0.57–0.86	**0** **.** **001**
**Step 2**	R^2^ Nagelkerke = 0.481			R^2^ Nagelkerke = 0.481		
Employment	0.84	0.76–0.94	**0** **.** **002**	0.88	0.81–0.95	**0** **.** **001**
Education	1.13	0.98–1.29	0.089	0.95	0.85–1.06	0.388
Alcohol use	1.04	0.88–1.22	0.674	1.06	0.93–1.21	0.398
Cannabis use	0.80	0.67–0.96	**0** **.** **016**	0.77	0.67–0.89	**<0** **.** **001**
Opioid use	0.75	0.55–1.02	0.070	0.77	0.62–0.96	**0** **.** **018**
Life satisfaction	2.14	1.94–2.36	**<0** **.** **001**	2.20	2.04–2.38	**<0** **.** **001**
Social confidants	1.61	1.36–1.90	**<0** **.** **001**	1.35	1.20–1.52	**<0** **.** **001**
**Step 3**	R^2^ Nagelkerke = 0.482			R^2^ Nagelkerke = 0.484		
Employment	0.85	0.76–0.94	**0** **.** **003**	0.88	0.81–0.95	**0** **.** **001**
Education	1.13	0.98–1.30	0.085	0.95	0.85–1.06	0.356
Alcohol use	1.54	0.70–3.32	0.277	1.02	0.56–1.85	0.951
Cannabis use	0.73	0.37–1.39	0.346	0.37	0.19–0.69	**0** **.** **002**
Opioid use	0.97	0.22–4.08	0.970	0.91	0.41–1.93	0.801
Life satisfaction	2.25	1.97–2.57	**<0** **.** **001**	2.16	1.96–2.38	**<0** **.** **001**
Social confidants	1.53	1.19–2.97	**0** **.** **001**	1.30	1.09–1.56	**0** **.** **004**
Alcohol*Life satisfaction	0.94	0.86–1.03	0.207	1.00	0.92–1.07	0.895
Alcohol*Social confidants	1.04	0.88–1.24	0.618	1.04	0.91–1.19	0.545
Cannabis*Life satisfaction	1.02	0.94–1.11	0.676	1.09	1.01–1.18	**0** **.** **031**
Cannabis *Social confidants	0.97	0.82–1.15	0.763	1.05	0.90–1.23	0.511
Opioid*Life satisfaction	0.96	0.81–1.14	0.607	0.99	0.90–1.10	0.860
Opioid*Social confidants	1.06	0.78–1.46	0.694	0.95	0.77–1.18	0.650

##### 3.2.1.2. Females

**Figure 1:**
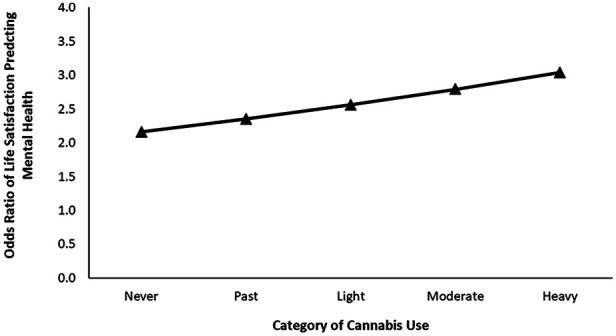
Odds ratio of life satisfaction predicting self-reported mental health at each level of cannabis use for females reporting low shame surrounding substance.

For females reporting low levels of shame towards general substance use, cannabis and opioid use significantly correlated with mental health (see Table 2). Increase in one level of frequency in cannabis use was associated with 24% increase in the likelihood of reporting poorer mental health (OR = 0.76, *p* < .001). Increase in one level of opioid use was associated with 30% increase in the likelihood of reporting poorer mental health (OR = 0.70, *p* = .001). Step 2 of this model revealed that, accounting for substance use and the covariates, higher levels of life satisfaction and social confidants were associated with increased odds of reporting better mental health (life satisfaction: OR = 2.20, *p* < .001; social confidants: OR = 1.35, *p* < .001). As in Step 1, both cannabis and opioid use remained to be significantly associated with mental health reporting (Cannabis: OR = 0.77, *p* < .001; Opioid: OR = 0.77, *p *= .018). In step 3 of this model, we also observed a significant relationship between cannabis use (OR = 0.37, *p *= .002), life satisfaction (OR = 2.16, *p *< .001), and social confidants (OR = 1.30, *p *= .004) with mental health. Importantly, life satisfaction significantly interacted with cannabis use to influence mental health ratings, OR = 1.09, *p* = .031. Tests of simple slope at each level of cannabis use revealed that the positive association between life satisfaction and mental health ratings increased sequentially as a function of greater cannabis consumption (see Figure 1). The odds ratios of life satisfaction was 2.16 for people who never used cannabis [95% CI (1.96, 2.38), *p* < .001], 2.35 for previous users of cannabis [95% CI (2.09, 2.64), *p* < .001], 2.56 for light users [95% CI (2.16, 3.04), *p* < .001], 2.79 for moderate users [95% CI (2.19, 3.55), *p* < .001], and 3.04 for heavy users (95% CI [2.22, 4.16], *p* < .001. No other interaction terms were significant.

#### 3.2.2. People reporting higher levels of shame towards substance use

##### 3.2.2.1. Males

For males reporting high levels of shame towards general substance use, alcohol, cannabis, and opioid use were significantly associated with mental health (see Table 3). Increase in one level of frequency of alcohol use was associated with a 15% increase in the odds of reporting poorer mental health (OR = 0.85, *p* < .001). Increase in one level of frequency of cannabis use was associated with a 25% increase in the odds of reporting poorer mental health (OR = 0.75, *p* < .001). One level increase in frequency of opioid use was associated with 33% increase in disclosing poorer mental health (OR = 0.67, *p* < .001). Accounting for substance use and the covariates in Step 2 of this model, life satisfaction and social confidants significantly predicted better mental health. Specifically, the odds of reporting better mental health were 2.03 times higher for each unit increase in life satisfaction (OR = 2.03, *p* < .001). While, the odds of reporting better mental health were 1.29 times higher for each unit increase in social confidants (OR = 1.29, *p* < .001). Moreover, both alcohol (OR = 0.90, *p* = .024) and cannabis (OR = 0.77, *p* < .001) remained to be significantly correlated with mental health reporting as in Step 1; however, opioid use was no longer significantly associated with our outcome. In Step 3 of this model, life satisfaction (OR = 1.93, *p* < .001) and social confidants (OR = 1.32, *p *= .007) also significantly correlated with one's perceived mental health. Contrary to our prediction, the social determinants of well-being did not significantly interact with substance use to influence mental health rating for this sub-group.

**Table 3:** Regression model for people reporting high feelings of shame towards substance us*e.*

	Male			Female		
Predictors	OR	95% CI	*p*	OR	95% CI	*p*
**Step 1**	R^2^ Nagelkerke = 0.093			R^2^ Nagelkerke = 0.061		
Employment	0.92	0.85–0.99	**0** **.** **028**	0.95	0.87–1.03	0.181
Education	1.03	0.93–1.14	0.557	1.00	0.90–1.11	0.960
Alcohol use	0.85	0.78–0.92	**<0** **.** **001**	0.94	0.85–1.04	0.244
Cannabis use	0.75	0.69–0.82	**<0** **.** **001**	0.76	0.69–0.83	**<0** **.** **001**
Opioid use	0.67	0.55–0.82	**<0** **.** **001**	0.71	0.59–0.86	**<0** **.** **001**
**Step 2**	R^2^ Nagelkerke = 0.478			R^2^ Nagelkerke = 0.515		
Employment	0.89	0.82–0.96	**0** **.** **003**	0.92	0.84–1.00	**0** **.** **049**
Education	1.02	0.92–1.13	0.752	0.92	0.82–1.03	0.172
Alcohol use	0.90	0.83–0.99	**0** **.** **024**	0.94	0.84–1.04	0.225
Cannabis use	0.77	0.71–0.84	**<0** **.** **001**	0.79	0.72–0.87	**<0** **.** **001**
Opioid use	0.83	0.67–1.02	0.071	0.89	0.73–1.08	0.246
Life satisfaction	2.03	1.89–2.17	**<0** **.** **001**	2.09	1.94–2.26	**<0** **.** **001**
Social confidants	1.29	1.14–1.46	**<0** **.** **001**	1.53	1.34–1.75	**<0** **.** **001**
**Step 3**	R^2^ Nagelkerke = 0.480			R^2^ Nagelkerke = 0.520		
Employment	0.89	0.82–0.96	**0** **.** **003**	0.92	0.85–1.00	0.061
Education	1.01	0.91–1.12	**0** **.** **829**	0.92	0.82–1.03	0.141
Alcohol use	0.81	0.58–1.11	0.189	0.66	0.45–0.97	**0** **.** **037**
Cannabis use	0.73	0.52–1.01	0.061	0.77	0.55–1.08	0.134
Opioid use	0.60	0.28–1.25	0.186	0.76	0.39–1.45	0.406
Life satisfaction	1.93	1.75–2.14	**<0** **.** **001**	2.04	1.83–2.27	**<0** **.** **001**
Social confidants	1.32	1.08–1.61	**0** **.** **007**	1.34	1.08–1.67	**0** **.** **009**
Alcohol*Life satisfaction	1.02	0.98–1.06	0.322	1.03	0.99–1.09	0.174
Alcohol*Social confidants	0.97	0.88–1.08	0.615	1.05	0.94–1.18	0.361
Cannabis*Life satisfaction	1.02	0.97–1.06	0.500	1.00	0.96–1.05	0.885
Cannabis *Social confidants	0.97	0.88–1.07	0.517	1.00	0.89–1.11	0.936
Opioid*Life satisfaction	1.00	0.91–1.11	0.963	0.96	0.88–1.05	0.384
Opioid*Social confidants	1.20	0.96–1.51	0.104	1.28	1.03–1.59	**0** **.** **027**

##### 3.2.2.2. Females

**Figure 2:**
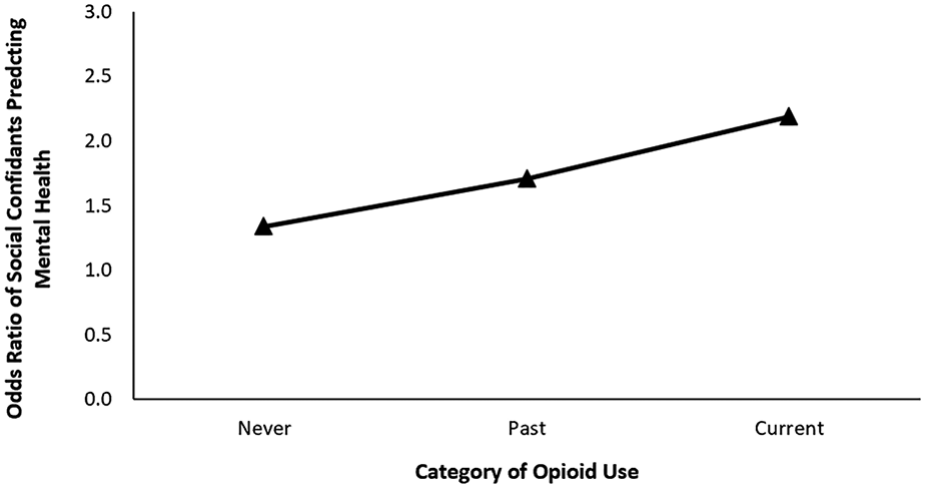
Odds ratio of social confidants predicting self-reported mental health at each level of opioid use for females reporting high shame surrounding substance use.

For females reporting high levels of shame towards substance use in general, increase in frequency of cannabis use was significantly associated with 24% increase in the odds of reporting poorer mental health (OR = 0.76, *p* < .001). Increase in one level of frequency of opioid use was also significantly associated with 29% increase in the odds of reporting poorer mental health (OR = 0.71, *p* < .001). Alcohol did not significantly influence mental health (*p* = .244). Controlling for substance use and the covariates in Step 2 of our model, higher ratings of life satisfaction (OR = 2.09, *p* < .001) and social confidants (OR = 1.53, *p* < .001) were significantly associated with increased likelihood of reporting better mental health ratings. Cannabis remained to be significantly associated with mental health scores (OR = 0.79, *p* < .001), but not opioid use (*p* =* *.246). In Step 3, we also observed that life satisfaction (OR = 2.04, *p *< .001) and social confidants (OR = 1.34, *p *= .009) were significantly correlated with the outcome measure. Importantly, social confidants significantly interacted with opioid use to influence mental health, OR = 1.28, *p* = .027. That is, for females who reported higher feelings of shame towards substance use, we found the greatest association between social confidants and mental health ratings in current opioid users, in comparison to past and non-users (see Figure 2). Specifically, the odds of ratio of social confidants increased from 1.34 [95% CI (1.08, 1.67), *p *= .009] for those who never used opioids, to 1.71 [95% CI (1.32, 2.23), *p* < .001] for previous users, and 2.19 [95% CI (1.41, 3.37), *p* < .001] for current users.

## 4. Discussion

The COVID-19 pandemic has had a profound influence on the social, economic, and health policies governing the Canadian population (11). Government restrictions put in place to contain the transmission of the virus have isolated individuals both physically and socially from family, friends, and other social support networks, resulting in feelings of loneliness and psychological distress (3). Further, past work has shown a global increase in recreational drug use during the COVID-19 pandemic (12). However, to date, there are limited studies examining the influence of substance use on the mental health ratings of a large Canadian sample during COVID-19, especially considering the correlates of well-being such as life satisfaction and social confidants. The current study addressed these research gaps by analyzing the “Canadian Perspectives Survey (CPSS) Series 6, 2021: Substance Use and Stigma During the Pandemic” dataset from Statistics Canada to examine the influence of substance use on mental health (16). Specifically, we examined whether substance use measured by frequency of alcohol, cannabis, and opioids use significantly predicted one's subjective reporting of mental health. In line with previous studies, we found that greater substance use was significantly associated with increased odds of reporting poorer mental health [(33); see (17) for review], for both males and females. Specifically, for males reporting low levels of shame towards substance use, we observed that only greater use of cannabis was significantly associated with poorer mental health. Further, greater life satisfaction and number of social confidants were associated with increased odds of reporting better mental health as suggested by past work (25), but did not significantly interact with substance use to influence mental health ratings. For females disclosing low shame surrounding substance use, greater use of cannabis and opioid significantly correlated with higher odds of reporting poorer mental health. Critically, our results revealed a significant interaction between life satisfaction and substance use in predicting mental health ratings. We found that as frequency of cannabis use increased, life satisfaction has a much greater positive association with mental health. Such a significant finding found only in females, but not in males, highlights a critical sex difference when considering the benefit of social and contextual factors on mental health. For males reporting high levels of shame, greater frequency of alcohol, cannabis, and opioid use were all significantly associated with greater odds of reporting poorer mental health. Moreover, both life satisfaction and social confidants did not significantly interact with substance use to predict mental health rating for this sub-group. For females reporting high shame, we found that greater frequency of cannabis and opioid use were associated with increased odds of reporting poorer mental health. Importantly, in this sub-group, maintaining social relations was particularly important to benefit the mental health of current users of opioids, relative to past and non-users.

As this was one of the few studies examining the influence of life satisfaction and social confidants on the association between substance use and mental health ratings during the context of COVID-19, we find it is critical to note that in females, social determinants exerted a greater influence on mental health than in males. This finding bolsters evidence to previous results suggesting differences in coping strategies used by both men and women (34). For example, past work shows that women experience internalizing symptoms such as feelings of despair, loneliness, and helplessness associated with mental health disorders (34). Whereas, males are shown to exhibit externalizing symptoms like excessive verbal and/or physical aggression, and involvement in socially deviant behaviors (34). We suggest that in females, the presence of greater social support and life satisfaction are important to diminish the internalizing symptoms associated with mental health disorders. Although in males, social determinants (life satisfaction and social confidants) failed to interact with substance use to influence mental health ratings in our data, we do believe social contextual correlates are still an important area of investigation for males. Future work should identity other critical constituents like family dynamic, parenthood, and occupation, predicted to greatly influence mental health of males (35).

The current study has a few limitations, mainly stemming from the lack of data that was available to us by Statistics Canada. First, no data was available on the purpose and nature of substance use. The substance use could be prescribed or un-prescribed. We acknowledge that the purpose, context, and nature of substance use can play a role in its relation to mental health. Similarly, data was only collected in participants aged 15 and up, and in the overall sample, only a few reported use of multiple substances, thus limiting our analyses from examining the influence of poly-drug use on perceived mental health. Future studies should assess how the use of multiple substances (i.e., poly drug use) may have an additive influence on mental health relative to the use of only one substance. The findings of the current study merely focused on the relation between the use of opioids, alcohol, and cannabis and mental health; thus, limiting the applicability of our findings to other types of substances. However, it is important to note here that these drugs are most commonly abused worldwide, and in Canada, due to its accessibility from commodification (36). Finally, shame surrounding substance use was measured as a general feeling towards the use of any substance. Therefore, we do not know whether self-reported ratings of shame would differ in relation to a particular type of substance use.

The results of the current study should be taken into consideration when evaluating the influence of substance use on mental health in males and females when developing treatment programs. It is critical that intervention programs direct attention to social determinants of mental health such as life satisfaction and the social network of the person who is under the influence of substances, to gain a comprehensive assessment of factors affecting one's mental health. Given that the transmission of COVID-19 continues to be a global health concern, the findings of this study offers valuable insight into areas that should receive attention from health and policy makers in order to reduce the mental health distress faced by the Canadian population. Specifically, we suggest investments into social services that offer resources at an individual level, such as career workshops, and focus groups that may help one to regain self-control and agency in their life and consequently report greater life satisfaction in the midst of an economic crisis (20). For example, females who express shame towards substance use may particularly benefit from social groups that will offer both social support and tangible resources to seek help and overcome maladaptive patterns of substance use. In addition, we also recommend community-building activities that instill a sense of social connection even in the presence of physical distancing. In conclusion, the results of this current study revealed that greater frequency of substance use was associated with poorer mental health ratings in both men and women; and crucially, at higher levels of substance use, the positive influence of life satisfaction and social confidants on mental health increased only for females.

## Data Availability

Publicly available datasets were analyzed in this study. This data can be found here: https://doi.org/10.17605/OSF.IO/APMQS.

## Footnotes

^1^Persons living on reserves, Aboriginal settlements, and households in extremely remote areas with very low population density were excluded from the survey.

## References

[1] Brooks SK Webster RK Smith LE Woodland L Wessely S Greenberg N The psychological impact of quarantine and how to reduce it: rapid review of the evidence. Lancet. (2020) 395(10227):912–20. 10.1016/S0140-6736(20)30460-8 32112714 PMC7158942

[2] Petzold MB Bendau A Plag J Pyrkosch L Mascarell Maricic L Betzler F Risk, resilience, psychological distress, and anxiety at the beginning of the COVID-19 pandemic in Germany. Brain Behav. (2020) 10(9):1745. 10.1002/brb3.1745 PMC7361063 32633464

[3] Zhang J Lu H Zeng H Zhang S Du Q Jiang T The differential psychological distress of populations affected by the COVID-19 pandemic. Brain Behav Immun. (2020) 87(April):49–50. 10.1016/j.bbi.2020.04.031 32304883 PMC7156946

[4] Wang C Pan R Wan X Tan Y Xu L Ho CS Immediate psychological responses and associated factors during the initial stage of the 2019 coronavirus disease (COVID-19) epidemic among the general population in China. Int J Environ Res Public Health. (2020) 17:1729. 10.3390/ijerph17051729 32155789 PMC7084952

[5] Cao W Fang Z Hou G Han M Xu X Dong J The psychological impact of the COVID-19 epidemic on college students in China. Psychiatry Res. (2020) 287:1–5. 10.1016/j.psychres.2020.112934 32229390 PMC7102633

[6] Forte G Favieri F Tambelli R Casagrande M. The enemy which sealed the world: effects of the COVID-19 diffusion on the psychological state of the Italian population. J. Clin. Med. (2020) 9:1802. 10.3390/jcm99061802 32531884 PMC7356935

[7] González-Sanguino C Ausín B Castellanos MÁ Saiz J López-Gómez A Ugidos C Mental health consequences during the initial stage of the 2020 coronavirus pandemic (COVID-19) in Spain. Brain Behav Immun. (2020) 87:172–6. 10.1016/j.bbi.2020.05.040 32405150 PMC7219372

[8] Bäuerle A Graf J Jansen C Dörrie N Junne F Teufel M An e-mental health intervention to support burdened people in times of the COVID-19 pandemic: CoPE It. J Pub Health. (2020) 42(3):647–8. 10.1093/pubmed/fdaa058 PMC7239146 32364242

[9] Varshney M Parel JT Raizada N Sarin SK. Initial psychological impact of COVID-19 and its correlates in Indian community: an online (FEEL-COVID) survey. Plos one. (2020) 15(5):233–874. 10.1371/journal.pone.0233874 PMC7259495 32470088

[10] Liu CH Zhang E Wong GTF Hyun S. Factors associated with depression, anxiety, and PTSD symptomatology during the COVID-19 pandemic: clinical implications for US young adult mental health. Psychiatry Res. (2020) 290:113–72. 10.1016/j.psychres.2020.113172 PMC7263263 32512357

[11] Robillard R Daros AR Phillips JL Porteous M Saad M Pennestri MH Emerging new psychiatric symptoms and the worsening of Pre-existing mental disorders during the COVID-19 pandemic: a Canadian multisite study: nouveaux symptômes psychiatriques émergents et détérioration des troubles mentaux préexistants durant la pandémie de la COVID-19: une étude canadienne multisite. Can J Psychiatry. (2021) 66(9):815–26. 10.1177/0706743720986786 33464115 PMC8504288

[12] Solomou I Constantinidou F. Prevalence and predictors of anxiety and depression symptoms during the COVID-19 pandemic and compliance with precautionary measures: age and sex matter. Int J Environ Res Public Health. (2020) 17(14):1–19. 10.3390/ijerph17144924 PMC7400373 32650522

[13] Bartel SJ Sherry SB Stewart SH. Self-isolation: a significant contributor to cannabis use during the COVID-19 pandemic. Subst Abus. (2020) 41(4):409–12. 10.1080/08897077.2020.1823550 33044893

[14] Kopel J Perisetti A Roghani A Aziz M Gajendran M Goyal H. Racial and gender-based differences in COVID-19. Front Public Health. (2020) 8:418. 10.3389/fpubh.2020.00418 32850607 PMC7399042

[15] Becker JB Hu M. Sex differences in drug abuse. Front Neuroendocrinol. (2008) 29(1):36–47. 10.1016/j.yfrne.2007.07.003 17904621 PMC2235192

[16] Statistics Canada. Canadian Perspectives survey series 6, 2021: substance use and stigma during the pandemic study documentation. Ottawa, Canada: Statistics Canada (2021).

[17] Drake RE Brunette MF. Complications of severe mental illness related to alcohol and drug use disorders. Recent Dev Alcohol. (1998) 14:285–99. 10.1007/0-306-47148-5_12 9751950

[18] Zaami S Marinelli E Varì MR. New trends of substance abuse during COVID-19 pandemic: an international perspective. Front Psychiatry. (2020) 11:1–4. 10.3389/fpsyt.2020.00700 32765328 PMC7378810

[19] World Health Organization. Overview of public health and social measures in the context of COVID-19. Geneva, Switzerland: World Health Organization (2020). 1–8.

[20] Rogowska AM Kuśnierz C Ochnik D. Changes in stress, coping styles, and life satisfaction between the first and second waves of the COVID-19 pandemic: a longitudinal cross-lagged study in a sample of university students. J Clin Med. (2021) 10(17):4025. 10.3390/jcm10174025 PMC8432555 34501473

[21] Diener E. New findings and future directions for subjective well-being research. Am Psychol. (2012) 67:590–7. 10.1037/a0029541 23163434

[22] López-Ortega M Torres-Castro S Rosas-Carrasco O. Psychometric properties of the satisfaction with life scale (SWLS): secondary analysis of the Mexican health and aging study. Health Qual. Life Outcomes. (2016) 14:170. 10.1186/s12955-016-0573-9 27938407 PMC5148901

[23] Proctor CL Linley PA Maltby J. Youth life satisfaction: a review of the literature. J Happiness Stud. (2009) 10(5):583–630. 10.1007/s10902-008-9110-9

[24] Mazza C Ricci E Biondi S Colasanti M Ferracuti S Napoli C A nationwide survey of psychological distress among Italian people during the COVID-19 pandemic: immediate psychological responses and associated factors. Int J Environ Res Public Health. (2020) 17(9):1–14. 10.3390/ijerph17093165 PMC7246819 32370116

[25] Stavrova O Luhmann M. Social connectedness as a source and consequence of meaning in life. J Posit Psychol. (2016) 11(5):470–9. 10.1080/17439760.2015.1117127

[26] Rahim M Patton R. The association between shame and substance use in young people: a systematic review. PeerJ. (2015) 2015:1. 10.7717/peerj.737 PMC4312064 25649509

[27] Abramowitz A Berenbaum H. Emotional triggers and their relation to impulsive and compulsive psychopathology. Pers Individ Dif. (2007) 43(6):1356–65. 10.1016/j.paid.2007.04.004

[28] O’Connor LE Berry JW Inaba D Weiss J Morrison A. Shame, guilt, and depression in men and women in recovery from addiction. J Subst Abuse Treat. (1994) 11(6):503–10. 10.1016/0740-5472(94)90001-9 7884834

[29] Vaske JJ Beaman J Sponarski CC. Rethinking internal consistency in Cronbach's alpha. Leis Sci. (2017) 39(2):163–73. 10.1080/01490400.2015.1127189

[30] Venables WN Ripley BD. Modern applied statistics with S. 4 edn. New York: Springer (2002). ISBN 0-387-95457-0, https://www.stats.ox.ac.uk/pub/MASS4/

[31] R Core Team. R: A language and environment for statistical computing. Vienna, Austria: R Foundation for Statistical Computing (2022). https://www.R-project.org/

[32] Yee TW. The VGAM package for categorical data analysis. J Stat Softw. (2010) 32(10):1–34. 10.18637/jss.v032.i10

[33] Dumas TM Ellis W Litt DM. What does adolescent substance use Look like during the COVID-19 pandemic? Examining changes in frequency, social contexts, and pandemic-related predictors. J Adolesc Health. (2020) 67(3):354–61. 10.1016/j.jadohealth.2020.06.018 32693983 PMC7368647

[34] Schrock D, Knop B. Gender and Emotions. In: Stets JE, Turner JH, editors. Handbook of the Sociology of Emotions. (Vol. 2). Dordrecht, Netherlands: Springer (2014). pp. 411–428.

[35] Affleck W, Carmichael V, Whitley R. Men's mental health: Social determinants and implications for services. *Can J Psychiatry*. (2018) 63(9):581–89. 10.1177/0706743718762388 PMC6109884 29673270

[36] Mota P. Avoiding a new epidemic during a pandemic: the importance of assessing the risk of substance use disorders in the COVID-19 era. Psychiatry Res. (2020) 290:113–42. 10.1016/j.psychres.2020.113142 PMC7253943 32502828

